# Steam Engine

**Published:** 1862-12

**Authors:** 


### STEAM ENGINE.

We propose in the next number of the Register to give a description and an engraving of a little rotary engine, in use in our laboratory, for running dental lathes; it is a gem. T.

